# A De Novo Mutation in *COL1A1* in a Holstein Calf with Osteogenesis Imperfecta Type II

**DOI:** 10.3390/ani11020561

**Published:** 2021-02-20

**Authors:** Joana G. P. Jacinto, Irene M. Häfliger, Fintan J. McEvoy, Cord Drögemüller, Jørgen S. Agerholm

**Affiliations:** 1 Department of Veterinary Medical Sciences, University of Bologna, 40064 Ozzano Emilia, Italy; joana.goncalves2@studio.unibo.it; 2 Institute of Genetics, Vetsuisse Faculty, University of Bern, 3012 Bern, Switzerland; irene.haefliger@vetsuisse.unibe.ch; 3 Department of Veterinary Clinical Sciences, University of Copenhagen, Dyrlægevej 16, DK 1870 Copenhagen, Denmark; fme@sund.ku.dk (F.J.M.); jager@sund.ku.dk (J.S.A.)

**Keywords:** cattle, *Bos taurus*, collagenopathy, skeletal disorder, bone disease, rare diseases, precision medicine, whole-genome sequencing

## Abstract

**Simple Summary:**

Skeletal connective tissue disorders represent a heterogeneous group of inherited disorders mostly monogenically inherited. Heritable connective tissue disorders such as osteogenesis imperfecta (OI) belong to this group. Herein, an affected calf showing congenital bone lesions such as intrauterine fractures, abnormally shaped long bones and localized arthrogryposis resembling OI type II is reported. Whole-genome sequencing (WGS) identified a most likely disease-causing mutation in the *COL1A1* gene. The *COL1A1* gene is known to be associated with dominant inherited OI type II forms in humans and sporadically in dogs and cattle, but so far, a variant in the fibrillar collagen NC1 domain has not been shown to cause a similar phenotype in domestic animals. We assume that the herein identified most-likely causative variant occurred either within the parental germlines or post-zygotically in the developing embryo. Rare lethal disorders such as OI in livestock are usually not diagnosed to the molecular level, mainly because of the lack of resources and diagnostic tools. WGS-based precision diagnostics allows understanding rare disorders and supports the value of surveillance of cattle breeding populations for harmful genetic disorders.

**Abstract:**

Osteogenesis imperfecta (OI) type II is a genetic connective tissue disorder characterized by bone fragility, severe skeletal deformities and shortened limbs. OI usually causes perinatal death of affected individuals. OI type II diagnosis in humans is established by the identification of heterozygous mutations in genes coding for collagens. The purpose of this study was to characterize the pathological phenotype of an OI type II-affected neonatal Holstein calf and to identify the causative genetic variant by whole-genome sequencing (WGS). The calf had acute as well as intrauterine fractures, abnormally shaped long bones and localized arthrogryposis. Genetic analysis revealed a private heterozygous missense variant in *COL1A1* (c.3917T>A) located in the fibrillar collagen NC1 domain (p.Val1306Glu) that most likely occurred de novo. This confirmed the diagnosis of OI type II and represents the first report of a pathogenic variant in the fibrillar collagen NC domain of *COL1A1* associated to OI type II in domestic animals. Furthermore, this study highlights the utility of WGS-based precise diagnostics for understanding congenital disorders in cattle and the need for continued surveillance for rare lethal genetic disorders in cattle.

## 1. Introduction

Osteogenesis imperfecta (OI) encompasses a heterogeneous group of rare genetic connective tissue disorders characterized by skeletal abnormalities, leading to bone fragility, deformity, low bone mass and growth deficiency [1,2]. Decrease bone strength predisposes to low-trauma factures or factures in atypical regions [3]. In humans, extra-skeletal manifestations of OI may include joint hypermobility, dentinogenesis imperfecta, blue sclera, hearing loss, and more rarely pulmonary and cardiovascular complications, and muscle weakness [2].

Currently, OI in humans is classified into as many as 18 types; the classification depends on the genetic causes, severity and clinical observation [4]. OI types I-IV are mainly associated with autosomal dominant variants in *COL1A1* and *COL1A2*; OI type V is a less frequent dominantly inherited form associated with variants in the novel gene *IFTM5*. The remaining types of OI, which usually arise at much lower frequency are autosomal recessive diseases while OI type XVIII has an X-linked inheritance pattern [2,4].

Since the second half of the 20th century several forms of OI have been reported in domestic animal species, including sheep [5], cats [6], dogs [7] and cattle [8]. Until now, one OI-related causative dominant variant in the *COL1A1* is known in dogs (OMIA 002126-9615) [9] and two in cattle (OMIA 002127-9913) [10,11]. In dogs, three OI-related causative dominant variants in the *COL1A2* are also known (OMIA 002112-9615) [12,13,14] and also in this species, an OI-related recessive form has been associated with a missense variant in *SERPINH1* (OMIA 001483-9615) [15].

In this study, we aimed to characterize an OI-affected Holstein calf, and to identify the causative genetic variant associated with the disorder using whole-genome sequencing (WGS).

## 2. Materials and Methods

### 2.1. Pathological Investigation

A male Holstein calf with a weight of 30.6 kg was delivered at gestation day 264 (normal gestation 281 days (mean)). The pregnancy was the result of insemination with semen of a purebred Holstein sire on a Holstein dam. The parents were not related within at least four generations. The calf was immediately humanely euthanized by an intravenous overdose of barbiturate upon delivery due to severe malformations. The carcass was submitted for necropsy during which radiographs were taken of the limbs. Tissue samples were taken during the necropsy and included internal organs, brain, metacarpus, metatarsus, humerus and tibia. These were fixed in 10 % neutral buffered formalin. Bone specimens were thereafter transferred to an aqueous solution containing sodium formate (0.5 mol/L) and formic acid (0.5 mol/L) (Kristensen’s decalcifying medium) until suitable for cutting. The tissues were thereafter trimmed, processed by routine methods, paraffin embedded, sectioned at 2 µm and stained with haematoxylin and eosin.

### 2.2. DNA Samples

Genomic DNA was extracted from skin and cartilage taken from the ear of the calf, from EDTA blood of its dam and from semen of its sire using Promega Maxwell RSC DNA system (Promega, Dübendorf, Switzerland).

### 2.3. Whole-Genome Sequencing

Using genomic DNA of the affected calf an individual PCR-free fragment library with approximately 400 bp inserts was prepared and sequenced for 150 bp paired-end reads using the NovaSeq6000 system (Illumina, San Diego, CA, USA). The sequenced reads were mapped to the ARS-UCD1.2 reference genome resulting in an average read depth of approximately 18.1× [16], and single-nucleotide variants (SNVs) and small indel variants were called. The applied software and steps to process fastq files into binary alignment map (BAM) and genomic variant call format (GVCF) files were in accordance with the 1000 Bull Genomes Project processing guidelines of run 7 (www.1000bullgenomes.com (accessed on 31 August 2018)) [17], except for the trimming, which was performed using fastp [18]. Further preparation of the genomic data had been done according to Häfliger et al. 2020 [19]. The impact of the called variants was functionally annotated with snpeff v4.3 [20], using the NCBI Annotation Release 106 (https://www.ncbi.nlm.nih.gov/genome/annotation_euk/Bos_taurus/106/ (accessed on 31 August 2018)), which resulted in the final VCF file, including all individual variants and their functional annotations. In order to find private variants, we compared the genotypes of the affected calf with 496 cattle genomes of various breeds that had been sequenced in the course of other ongoing studies and that are publicly available (Table S1) in the European Nucleotide Archive (SAMEA6528897 is the sample accession number of the affected calf; http://www.ebi.ac.uk/en (accessed on 7 February 2020)). Integrative Genomics Viewer (IGV) [21] software version 2.0 was used for visual inspection of genome regions containing possible candidate genes.

### 2.4. Targeted Genotyping

Polymerase chain reaction (PCR) and Sanger sequencing were used to validate and genotype the variant identified from WGS. The *COL1A1* missense variant (NM_001034039.2: g.36473359T>A) was genotyped using the following primers: 5′- ATCTTACTTTGCCCCACCCC-3′ (forward primer) and 5′-GGCTACAAGGTCCAG CTCAC-3′ (reverse primer). The sequence data were analyzed using Sequencher 5.1 software (GeneCodes, Ann Arbor, MI, USA).

### 2.5. Evaluation of the Molecular Consequences of Amino Acid Substitutions

PROVEAN [22] and DynaMut [23] were used to predict the functional consequences of the discovered variant on protein. For multispecies sequence alignments the following NCBI proteins accessions were used: NP_001029211.1 (*Bos taurus*), NP_000079.2 (*Homo sapiens*), XP_001169409.1 (*Pan troglodytes*), XP_001096194.2 (*Macaca mulatta*), NP_001003090.1 (*Canis lupus*), NP_031768.2 (*Mus musculus*), NP_445756.1 (*Rattus norvegicus*), NP_954684.1 (*Danio rerio*), NP_001011005.1 (*Xenopus tropicalis*).

### 2.6. Sequence Accessions

All references to bovine *COL1A1* gene correspond to the NCBI accessions NM_001034039.2 (*COL1A1* mRNA) and NP_001029211.1 (COL1A1 protein). For the protein, structure of COL1A1 the Uniprot database (https://www.uniprot.org/ (accessed on 31 August 2018)) accession number P02453 was used.

## 3. Results

### 3.1. Pathological Phenotype

**Figure 1:**
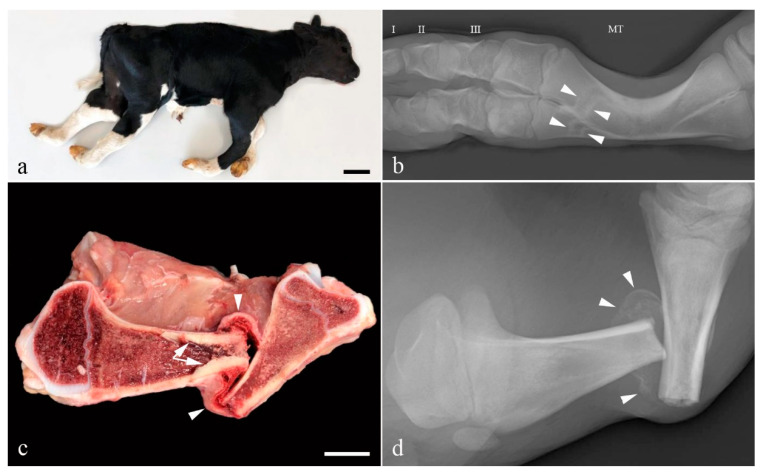
Gross morphology of the OI affected Holstein calf. (**a**) The limbs appear shortened and the distal joints are flexed. Bar = 10 cm; (**b**) Radiograph of a metatarsal bone (MT). The diaphysis is curved and transverse lines of sclerosis (arrow heads) are present distally. These may represent sites of healed fracture or growth arrest lines. Phalanx I, II and III are indicated by their respective numbers; (**c**) Longitudinal section through the left tibia. A transverse fracture with dislocation of the fracture ends is seen. Prominent osseous endostal proliferation has developed in the proximal part of the fracture (arrows). The fracture is surrounded by fibrosis (arrow heads). The width of the cortex is un-uniform with the caudal part of the proximal diaphysis/metaphysis being thin. Bar = 2.5 cm; (**d**) Radiograph of the specimen shown in (**c**) before longitudinal sectioning. The non-aligned fracture ends are surrounded by partly mineralized fibrous callus (arrow heads).

The limbs appeared shortened with bilateral symmetric flexion of the fetlock joint and mild lateral rotation of the digits. Flexion of the thoracic limb metacarpo-phalangeal joints was mild (15°) while it was almost 90° in the pelvic limbs. The tibiotarsal joints were extended (Figure 1a). Multiple long bones were abnormal and especially the metacarpal and metatarsal bones were bowed and appeared of reduced diameter (Figure 1b). Several transverse or oblique fractures, some with displaced fracture ends, were present in the long bones and the left hemimandible. Some fractures had fibrous callous formation and cortical bone proliferation. Non-aligned fracture ends showed abnormal healing (Figure 1c,d), while others were acute. Teeth appeared normal as did the color of the sclera.

**Figure 2:**
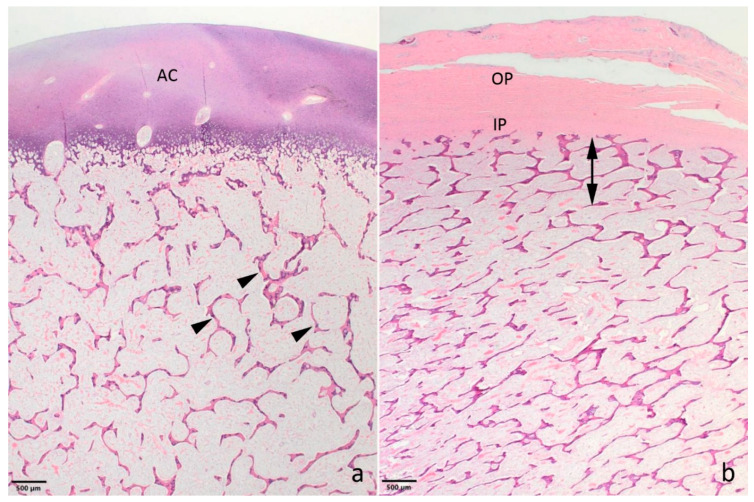
Photomicrographs showing the bone morphology of the OI affected Holstein calf. (**a**) The epiphyseal spongiosa is characterized by small trabeculae with remnants of chondroid matrix (arrow heads). The amount of spongious bone is reduced resulting in wide spaces between the trabeculae. AC: articular cartilage. Distal epiphysis, metacarpus. (**b**) The compacta (double headed arrow) is poorly developed and difficult to distinguish from the spongious bone. As for the epiphyses, the trabecular bone is poorly developed. IP and OP: inner and outer layer of the periost, respectively. Metaphysis, distal metatarsus. **a**,**b**: Haematoxylin and eosin.

Histology only revealed lesions in the bones. The epiphyseal trabeculae were reduced in size and number (Figure 2a). Also, ossification was reduced as chondroid matrix was widely present in bone spicules and occasional islets of chondroid matrix were seen. The epiphyseal growth lines were normal, but as for the epiphyses, the metaphyseal and diaphyseal trabecular bone was of reduced amount and quality. The cortical bone appeared thinner (Figure 2b). The bony ends of the fractured left tibia were completely covered by a prominent fibrous callus, while the fracture of the left metacarpus had no signs of repair.

### 3.2. Genetic Analysis

Filtering of WGS for private variants present in the affected calf and absent in the 496 available control genomes, identified 14 protein-changing variants with a predicted high and moderate impact on the encoded protein. They were found to be heterozygous exclusively in the OI-affected calf and absent in the 496 control genomes that were sequenced in the course of other ongoing projects at the Institute of Genetics. These variants were further investigated for their occurrence in a global control cohort of 3103 genomes of a variety of breeds 1000 Bull Genomes Project run 7 [17], which revealed five protein-changing variants exclusively present heterozygous in the genome of the affected calf (Table S2).

**Figure 3:**
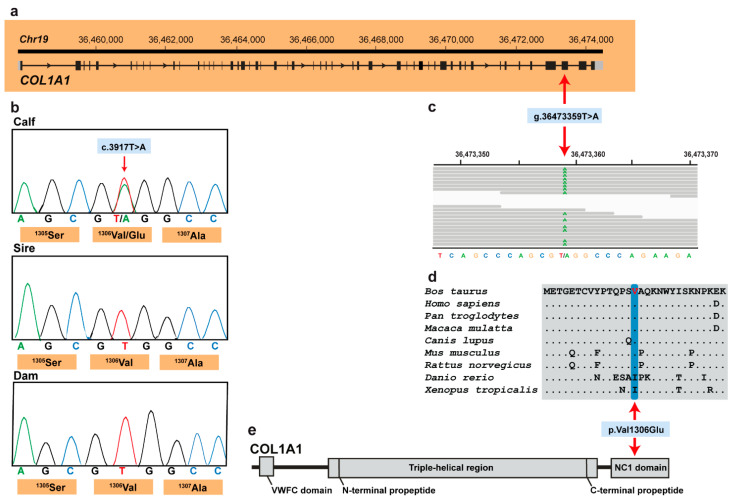
*COL1A1* missense variant in an OI type II-affected Holstein calf. (**a**) *COL1A1* gene structure showing the variant location on chromosome 19, exon 49 (red arrow); (**b**) Electropherograms showing the heterozygous genotype of the calf, and the absence of the variant in its dam genome and in the germline of its sire. (**c**) IGV screenshot presenting the Chr19: g.36473359T>A variant in the affected calf. (**d**) Multiple sequence alignment of the collagen alpha-1(I) chain of the COL1A1 protein encompassing the region of the p.Val1306Glu variant demonstrates complete evolutionary conservation across species. (**e**) Schematic representation of COL1A1 protein and its three functional domains.

These five variants were subsequently visually inspected using IGV software confirming all as true variants. Of all the remaining private variants, only one occurred in an obvious candidate gene for OI (Figure 3a). The heterozygous variant at chr19:36473359T>A represents a missense variant in COL1A1 (NM_001034039.2: c.3917T>A; Figure 3c). This variant alters the encoded amino acid of COL1A1 residue 1306 (XP_024835395.1: p.Val1306Glu) located in the fibrillar collagen NC1 domain (Figure 3d,e). Furthermore, the valine to glutamine substitution affects an evolutionary conserved amino acid (Figure 3d) and was predicted to be deleterious (PROVEAN score −4.96) and destabilizing (DynaMut, ΔΔG: −0.127 kcal/mol). To confirm and evaluate the presence of the COL1A1 variant, the affected genomic region was amplified by PCR and Sanger sequenced in the calf, its sire and dam (Figure 3b). Analyzing the sequencing data, we observed that the calf was indeed heterozygous for the detected COL1A1 variant whereas the sire and dam were both homozygous for the wild type allele (Figure 3b). This showed that the mutation most likley arose spontaneously in the affected calf and finally confirmed the diagnosis of OI type II.

## 4. Discussion

The identified missense variant in *COL1A1* in an obvious candidate gene represents the most likely pathogenic variant associated with the observed OI phenotype. As for humans, OI in cattle is a disorder characterized by bone fragility with perinatal fractures, severe bowing of long bones and reduced mineralization. Furthermore, the OI type II form is frequently lethal in utero or shortly after birth due to severe bone fragility and respiratory insufficiency. In cattle, forms of OI type II (OMIA 002127-9913) have been reported in Fleckvieh and Red Angus cattle associated to dominant acting pathogenic variants in *COL1A1* inherited from mosaic sires [10,11]. In humans, this disorder (OMIM 166210) is linked to pathogenic variants in *COL1A1* and *COL1A2* with a dominant pattern of inheritance. Thus, cases of OI with lesions typical for type II OI could be suspected of having a defect in *COL1A1* or *COL1A2* genes; a suspicion that is helpful when analysing WGS data. In this case, filtering for private variants lead to the identification of a missense variant in *COL1A1*. We assume that this spontaneous mutation most likely occurred either within the parental germlines or post-zygotically in the developing embryo. The mutant allele was detected neither in the dam nor in the sire, given that the variant was not found in the paternal germ line DNA which was analysed. This means that the missense variant was exclusively present in heterozygous state in the affected offspring only. Therefore, it appears more plausible that the identified mutation arose indeed de novo spontaneously in the very early development of the calf. However, a low level mosaicism in the dam cannot be excluded given that the DNA which was analysed was not from the germline.

The identified deleterious variant and the conservation of the affected amino acid residue of COL1A at the position 1306 suggest that this variant is most likely pathogenic. The affected valine residue is conserved across mammals and corresponds to isoleucine in more distant related species such as clawed frogs or zebrafish. As isoleucine and valine are highly similar amino acids having large aliphatic hydrophobic side chains their molecules are rigid, and their mutual hydrophobic interactions are important for the correct folding of proteins, as these chains tend to be located inside of the protein molecule. Because glutamic acid expressed by the mutant allele carries a long hydrophilic acidic group with strong negative charge it most likely impairs proper folding. Type I collagen is a member of group I collagen (fibrillar forming collagen) and is located in the extracellular matrix (ECM). There is a wide range of connective tissue disorders that occur from genetic abnormalities in ECM proteins as for example OI. In contrast to heterozygous null mutations, most of the more severe ECM disorders are caused by heterozygous missense mutations that interrupt the protein structure [24]. Variants in genes coding for collagens provide the perfect scenario for the impact of dominant negative protein structural mutations. In addition to variants in *COL1A1* and *COL1A2* associated with OI, many pathogenic variants in *COL2A1* and *COL10A1* are linked to chondrodysplasias [24].

Interestingly, the identified missense variant in this study occurred in the fibrillar collagen NC1 domain, and by analogy with substitutions in this domain, as in collagen alpha-10(X) in Schmid metaphysical chondrodysplasias (OMIM 156500), it would be expected to disrupt the collagen homotrimer assembly and secretion leading to proteosomal degradation of the unassembled collagen chains [25,26,27,28]. To the best of our knowledge, no pathogenic variant in fibrillar collagen NC1 domain of COL1A1 associated with OI has been reported in domestic animal species and therefore represents the first large animal model for mutations occurring in this domain of COL1A1.

## 5. Conclusions

This is the first report of a most likely pathogenic missense variant in OI type II-affected cattle affecting the fibrillar collagen NC domain of COL1A1. So far, disease-causing variants in *COL1A1* in domestic animals were found only in the triple-helical region of the encoded collagen protein. This case demonstrates that pathogenic variants in the other domains of the COL1A1 protein also cause a similar congenital phenotype. Therefore, this finding expands the spectrum of *COL1A1* mutations that cause a uniform phenotype. Furthermore, this example highlights the utility of WGS-precise diagnosis for understanding sporadic cases of congenital disorders associated to de novo mutations and the need for continued surveillance of genetic lethal disorders in cattle breeding.

## Data Availability

The whole-genome data of the affected calf is freely available at the European Nucleotide Archive (ENA) under sample accession number SAMEA6528897.

## Supplementary Materials

Supplementary materials can be found at https://www.mdpi.com/2076-2615/11/2/561/s1. Table S1: EBI Accession numbers of all publicly available genome sequences. We compared the genotypes of the calf with 494 cattle genomes of various breeds that had been sequenced in the course of other ongoing studies and that were publicly available, Table S2: List of the remaining variants after the comparison to the global control cohort of 3103 genomes of other breeds (1000 Bull Genomes Project run 7; www.1000bullgenomes.com (accessed on 31 August 2018)), revealing 5 protein-changing variants only present in the genome of the OI type II-affected calf. These 5 variants with a moderate predicted impact on the encoded protein were located within 5 different genes or loci. Note that the predicted pathogenic variant NM_001034039.2: c.3917T>A is the only one located in a candidate gene for bone diseases.
